# Profiles of cybervictimization among college students and associated psychosocial patterns: a latent profile and network analysis

**DOI:** 10.3389/fpsyg.2026.1922137

**Published:** 2026-10-06

**Authors:** Honglei Wang, Lixuan Zhuang, Xi Zhang, Tengfei Yu, Ting Liu, Xiuling Yang

**Affiliations:** School of Nursing, Qingdao University, Qingdao, Shandong, China

**Keywords:** college student, cybervictimization, latent profile analysis, network analysis, psychosocial factors

## Abstract

**Introduction:**

Cybervictimization has become a prevalent global public concern as it threatens the wellbeing of young people. Understanding the distinct patterns of cybervictimization, along with their associated factors is crucial for developing effective interventions.

**Methods:**

A total of 1,009 college students were selected from three comprehensive universities in Shandong Province, China, via convenience sampling. Participants completed online assessments of cybervictimization and related variables. Latent profile analysis was employed to classify the cybervictimization subgroups, and network analysis was used to explore the interaction of risk factors among different subgroups, identifying core risk factors.

**Results:**

The major results are summarized as follows: (1) The cybervictimization among college students were classified into three distinct subgroups: low cybervictimization group (48.3%), moderate cybervictimization group (38.2%), and high cybervictimization group (13.5%). (2) Network analysis revealed that School belonging was the primary core factor (strength/expected influence = 2.125/1.920). The low and moderate cybervictimization groups showed higher strength and expected influence in online comfort (low: 0.481/1.041, moderate: 0.389/0.931), while the high cybervictimization group had higher values in online contact risk and deficient impulse control (strength = 0.730/1.060). Furthermore, the Network Comparison Test (NCT) confirmed significant differences in symptom network structure between the low and high cybervictimization networks (*M* = 0.268, *p* < 0.05).

**Conclusion:**

This study identified three latent profiles of cybervictimization among college students and revealed both shared and profile-specific central factors, with school belonging central across all profiles and online contact risk and deficient impulse control particularly salient in the high-victimization group. These findings advance understanding of the psychosocial mechanisms underlying cybervictimization and offer preliminary, subgroup-informed insights for its prevention among college students.

## 1 Introduction

With continuous advancements in internet technology, online social networking has gradually become a critical avenue for interpersonal communication, offering new opportunities for individuals to fulfill their social needs. However, it has also increased their risk of exposure to online interpersonal aggression (Yang et al., 2023). When individuals become the actual target of such aggression, this experience is referred to as cybervictimization. Although cybervictimization can arise from various forms of online aggression, the present study focuses specifically on victimization stemming from cyberbullying, defined as the experience of being the target of intentional and repeated aggressive behaviors perpetrated through electronic communication, including verbal abuse, rumor spreading, intimidation, threats, extortion, and online exclusion (Dai et al., 2025). Multiple studies indicate that college students represent a typical group affected by cybervictimization, as they spend extended periods online and have relatively unrestricted access to the internet (Gupta et al., 2023; Xie et al., 2022). This is particularly evident in China, with the prevalence of cybervictimization among college students ranging from 32% to 64% (Huang et al., 2021; Zhang et al., 2026), comparable to the mid-to-high range reported in some developing countries (Al-Shatnawi et al., 2024; Alrajeh et al., 2021; Sheikh et al., 2023). As a serious adverse online experience, cybervictimization not only exerts negative effects on mental health but also increases the risk of various health-related risky behaviors, including non-suicidal self-injury, self-harm, aggression, violence, and smoking, or alcohol use (Arif et al., 2024; Bishop et al., 2023; Lee et al., 2025; Romualdo et al., 2025; Wang et al., 2020). Notably, the ongoing transformation of the digital landscape, particularly the rise of artificial intelligence (AI) technologies, has introduced new complexity to cybervictimization. Specifically, Pei et al. (2026) demonstrated that large language models (LLMs) can generate automated abusive messages that match or exceed human-authored aggression in perceived severity. Meanwhile, tools such as AI-driven image generators make it easier for malicious actors to turn ordinary social media photos into non-consensual explicit or defamatory content. Even without engaging in high-risk online behaviors, targets of AI-generated harassment may suffer from heightened psychological distress, reputation damage, and social difficulties (Flynn et al., 2025; Wahab, 2025). Given the severity and prevalence of cybervictimization, a growing body of empirical research has sought to identify the factors associated with this phenomenon.

Existing theoretical and empirical evidence suggests that cybervictimization is associated with psychosocial factors operating across multiple levels of the individual's surrounding environment. Ecological systems theory provides the overarching framework for organizing these environmental influences (Bronfenbrenner, 1979), with the microsystem, comprising the individual, family, peers, and school, exerting the most direct effect because interactions within this proximal layer shape psychological and behavioral development. Family functioning illustrates this influence directly. Adolescents from well functioning families manage external stressors more effectively (Çimen, 2018; Cui and Li, 2023), while childhood psychological abuse and dysfunctional parenting increase vulnerability to cybervictimization (Geng et al., 2022; Gómez-Ortiz et al., 2018). Beyond the family, the university environment constitutes another fundamental social domain, where perceptions of a negative school climate significantly predict cybervictimization risk (Harrison-Pavlik et al., 2024) and lower school connectedness, encompassing both interpersonal respect and psychological belonging, is strongly associated with heightened vulnerability (Menabò et al., 2024). Peer relationships show a similar pattern. Traditional peer victimization overlaps substantially with cybervictimization, with 46 percent of cybervictims in one study also reporting traditional victimization (Chudal et al., 2022), indicating that students already victimized offline face a heightened risk of similar difficulties online.

Ecological systems theory helps locate factors associated with cybervictimization within the family, peer, and school contexts surrounding the individual, but the theory offers limited insight into how these contextual factors are translated into actual cybervictimization experiences through online behavioral mechanisms. This translation from context to experience unfolds primarily at the level of the individual. Together, the frameworks introduced below address this individual-level process from three complementary angles, spanning cognitive vulnerability, behavioral exposure, and psychological state. The cognitive-behavioral model of pathological internet use specifies maladaptive cognition as a proximal cognitive vulnerability (Davis, 2001), which may drive individuals toward problematic internet use and internet addiction as risky online behaviors, both of which have been identified as important antecedents of cybervictimization (Tian et al., 2017). From an exposure perspective, cyber-routine activities theory further explains how such behaviors may increase exposure to specific cybervictimization experiences, proposing that cybervictimization arises from the convergence of three elements, namely a suitable target, the absence of a capable guardian, and a motivated offender (Choi and Lee, 2017). Within this framework, the extent to which an individual presents as a suitable target depends largely on their online lifestyle. Frequent interaction with strangers, excessive disclosure of personal information, and participation in contentious online discussions are concrete expressions of this risky online lifestyle, each associated with a higher risk of victimization (Antisdel, 2025; Choi et al., 2019). These behavioral factors primarily influence individuals' likelihood of exposure to cybervictimization, whereas individuals' psychological states may further shape the actual occurrence of cybervictimization experiences. The stress generation model proposes that psychological distress is not only a consequence of negative experiences but may also contribute to the occurrence of subsequent negative events (Chu et al., 2019; Smith et al., 2022). Depression, as a negative psychological state, has been identified as an important predictor of cybervictimization (Albikawi, 2023). One possible mechanism underlying this association is that depressed students are more inclined to seek social connection with strangers online, thereby increasing their exposure to victimization risk (Radovic et al., 2017). Individuals also differ in their capacity to regulate emotions following negative experiences, and this difference can be characterized by regulatory emotional self-efficacy (RESE). RESE, derived from self-efficacy theory (Caprara et al., 2008), reflects one's perceived capacity to regulate their own emotional states and has been identified as a key psychological resource for coping with adverse experiences such as victimization (Riley et al., 2019). Specifically, individuals with lower emotional self-efficacy are more prone to rumination and catastrophic interpretation when facing cybervictimization (Quintana-Orts et al., 2023; Schunk et al., 2022), and this perceived helplessness may signal vulnerability to potential offenders, marking the individual as an easier target and increasing the likelihood of cybervictimization.

Integrating these theoretical perspectives, the present study developed a comprehensive conceptual model encompassing depression, regulatory emotional self-efficacy, maladaptive cognition related to internet use, risky online behavior, peer victimization, family functioning, and school connectedness, as illustrated in Figure 1. These variables were selected to capture the individual, interpersonal, family, and school contexts potentially relevant to cybervictimization.

**Figure 1:**
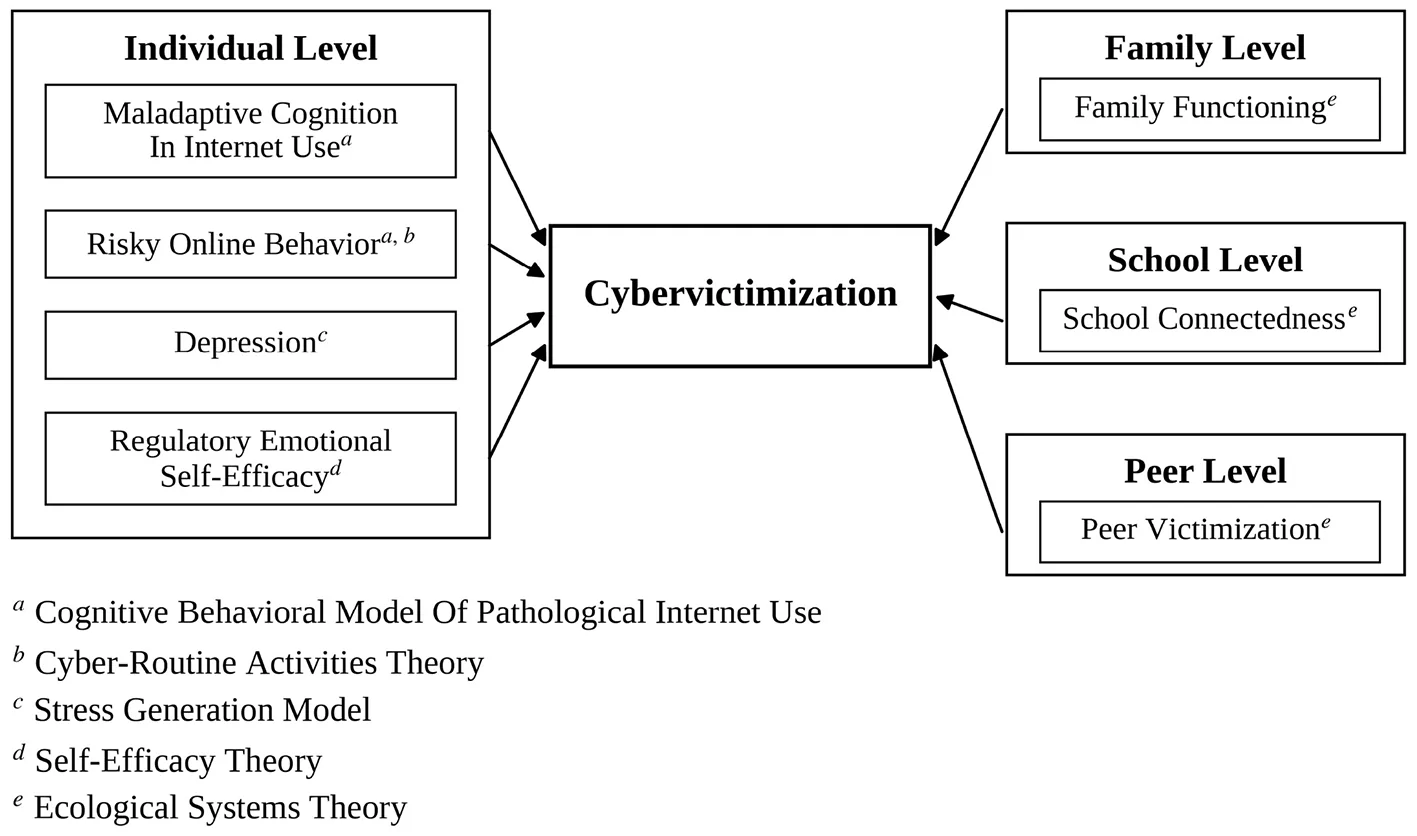
Predict variables related to cybervictimization.

Despite the identification of multiple psychosocial factors associated with cybervictimization, how these factors are interconnected and whether their patterns of association vary across individuals with different levels of cybervictimization remain unclear. One plausible reason may be that these studies employed a variable-centered approach to examine factors associated with cybervictimization, testing the unique contribution of each factor rather than their complex interactions. Ding et al. (2024) found that the relationship between cumulative risk factors and school bullying victimization followed a non-linear quadratic pattern, with victimization increasing more rapidly at first and then leveling off as the number of risk factors increased, suggesting that multiple risk factors may reinforce one another rather than simply exert additive effects. This finding echoes the view held by network approaches to psychological and behavioral phenomena, which propose that risk factors do not operate in isolation but instead form a dynamically interconnected system characterized by mutual interaction (Hevey, 2018). For example, maladaptive cognition of internet use, typically characterized by distorted beliefs about the self and the online environment along with avoidance of and helplessness toward offline social interaction, has been associated with elevated risky online behavior among cybervictimized individuals, which may further compound victimization risk (Qiu and Ye, 2024). Psychological difficulties such as anxiety and depression have similarly been linked to risky online behavior (Liu et al., 2019). However, prior research has not yet integrated these known pairwise associations into a unified statistical framework to capture the overall pattern of association among factors related to cybervictimization. This oversight is critical, as addressing it could improve our understanding of the role psychosocial factors play in the etiology of cybervictimization.

Network analysis provides a method for testing this overall pattern of association directly (Zhao et al., 2025). This method represents each factor as a node and the statistical association between factors as an edge connecting the nodes, thereby constructing a network structure that reflects how the factors interact with one another. Within this structure, factors that are connected to many other nodes with greater strength are identified as central nodes, and these factors play a key role in maintaining the overall network, such that intervening on central nodes may produce broader effects than intervening on a single isolated factor. Network analysis has been applied to examine the interplay of biopsychosocial factors and their influence on childhood harm. For example, Vial et al. (2020) found that risk factors for child maltreatment form an interconnected network, and that intervening on central factors may destabilize the overall risk network a nd thereby reduce the likelihood of maltreatment. Therefore, compared with traditional approaches, network analysis is particularly well suited for uncovering the relational patterns among these factors.

The network analysis presented above captures the average pattern of associations across the entire sample, reflecting the general co-occurrence of risk factors at the group level. However, prior research suggests that individuals classified as cybervictims may exhibit qualitative differences that network analysis alone cannot capture (Smokowski et al., 2014; Staude-Müller et al., 2012). Such differences may be reflected not only in the severity of cybervictimization but also in the underlying patterns of victimization experiences. To address this issue, latent profile analysis (LPA) has been increasingly applied to identify subgroups characterized by distinct symptom configurations (Chen et al., 2026; Ciminli, 2025). Rather than reporting sample-level averages as variable-centered approaches do, LPA classifies individuals according to their response patterns across scale items, with the final class solution determined jointly by statistical fit and interpretability (Nylund-Gibson et al., 2019). Therefore, before conducting network analysis of the psychosocial factors associated with cybervictimization, it is important to first identify distinct subgroups of cybervictimization, so as to gain deeper insight into the relationships and core features across these subgroups and to inform more effective and targeted intervention strategies (Luo et al., 2026). In summary, this study integrates LPA with network analysis to pursue three main objectives: First, what latent profiles can be identified from college students' cybervictimization experiences? Second, does the network structure of psychosocial factors differ across cybervictimization profiles? Third, which psychosocial factors show higher centrality within each cybervictimization profile and may warrant further consideration as potential intervention targets? By identifying these patterns, this study aims to provide preliminary evidence for future intervention research and to deepen the understanding of the associations among psychosocial factors involved in cybervictimization among college students.

## 2 Methods

### 2.1 Participants

This study employed a multistage convenience sampling approach. First, three comprehensive universities in Shandong Province were selected based on institutional accessibility and feasibility. Second, two academic majors were conveniently selected from each university, yielding six majors across three disciplinary domains: Medical Sciences (Nursing, Stomatology), Information Sciences (Computer Science and Technology, Communication Engineering), and Humanities and Social Sciences (Preschool Education, Teaching Chinese to Speakers of Other Languages). The selected majors covered diverse disciplinary backgrounds. Students across all grade levels within these six majors were invited to participate via an online questionnaire link between September and December 2025. The inclusion criteria were as follows: (1) age between 18 and 26 years, (2) cognitive ability to complete the questionnaire, and (3) voluntarily participate in this study and sign the informed consent form. The exclusion criteria included: (1) cognitive impairment or developmental delay, (2) severe mental disorders, (3) students on suspension or leave of absence, and (4) International students. A total of 1,125 responses were collected; after excluding invalid questionnaires, 1,009 valid responses remained, representing an effective rate of 89.7%. This study was conducted in strict accordance with ethical standards and approved by the Ethics Committee of the authors' University (QDU-HEC-2025495).

### 2.2 Measures

#### 2.2.1 Cybervitimization

The Cyberbullying Victims' Behaviors Scale, originally developed by Çetin et al. (2011) and translated and adapted into Chinese by You (2013), was used to assess the frequency of cybervictimization among college students over the past year. The Chinese version has been applied and psychometrically validated among Chinese college students, demonstrating satisfactory reliability (Cronbach's α = 0.825; split-half reliability = 0.747; subscale α = 0.613–0.806) and a three-factor structure consistent with the original scale (χ^2^/df = 2.608, CFI = 0.97, RMSEA = 0.06). The scale comprises 12 items across three dimensions: cyber verbal bullying, hidden identity, and cyber fabricate, rated on a 5-point Likert scale ranging from 1 (never) to 5 (always). In the present study, Cronbach's α for the total scale was 0.88.

#### 2.2.2 Depression

The Patient Health Questionnaire-9 developed by Kroenke et al. (2001) and revised by Zhang et al. (2013) was used to assessed depression. Comprising 9 items, the scale uses a 4-point Likert system (0 = not at all, 3 = nearly every day), resulting in a total score range of 0 to 27. Elevating scores indicate an increase in symptom severity. In this study, the Cronbach'α coefficient for the scale was 0.87.

#### 2.2.3 Regulatory emotional self-efficacy

Students' emotional self-regulation developed by Caprara et al. (2008) and revised by Wen et al. (2009) was measured using the Regulatory Emotional Self-efficacy Scale, which contains 12 items divided into three dimensions: managing anger (ANG), managing distress (DES), and expressing positive affect (POS). Each item employs a 5-point Likert response format (1 = strongly disagree, 5 = strongly agree). In this study, the tool showed excellent reliability, with a Cronbach'α coefficient of 0.884.

#### 2.2.4 Family functioning

Family functioning was evaluated using the Chinese version of Overall Family Functioning Scale (Zou et al., 2010). The scale includes 6 items measuring dimensions such as family atmosphere, family cohesion, and coping with difficulties. Items are rated on a 5-point Likert scale (1 = strongly disagree to 5 = strongly agree), where higher scores reflect a more favorable family environment. In this study, the internal consistency reliability was 0.932.

#### 2.2.5 School connectionness

Participants' connection to their educational institution was assessed using the Chinese version of School Connectedness Scale (Yu et al., 2011). This 10-item instrument covers three dimensions: peer support, teacher support, and school belonging. Items are rated on a 5-point Likert scale (1 = none to 5 = all), where higher scores reflect better-perceived school functioning. The Cronbach's α coefficient for this scale in the present study was 0.905.

#### 2.2.6 Peer victimization

Traditional peer victimization was measured using the Chinese version of Peer Victimization Questionnaire (Zhou et al., 2014). Comprising 9 items, the scale captures three forms of victimization: verbal, relational, and physical. Responses are based on a 5-point Likert system (1 = never, 5 = ≥ 4 times), with higher total scores signifying more frequent victimization experiences. In the present study, the scale's Cronbach's α coefficient was 0.879.

#### 2.2.7 Maladaptive cognition in internet use

The Online Cognition Scale developed by Davis et al. (2002) and revised by Liang (2008) was used to assess participants' maladaptive cognition in internet use. The scale includes 14 items across three dimensions: online comfort, diminished impulse control, and escape and withdrawal. Each item employs a 5-point Likert response format (1 = strongly disagree, 5 = strongly agree), where elevated scores reflect more severe maladaptive cognition. For the present sample, the scale demonstrated excellent reliability, with a Cronbach'α of 0.869.

#### 2.2.8 Online risky behavior

The Chinese version of Online Risky Behavior Scale was utilized to assess participants' activities and behaviors in the online environment (Qiu and Ye, 2024). Spanning 24 items, the scale assessed five subscales: entertainment risk, social networking risk, contact risk, economic transaction risk, and management risk. Using a 5-point Likert scale (1 = “strongly disagree”, 5 = “strongly agree”), greater overall scores signify a higher likelihood of engaging in online risky behaviors. For the present sample, the scale demonstrated excellent reliability (Cronbach'α = 0.875).

### 2.3 Quality control

To ensure data quality, several measures were implemented. All participants were thoroughly informed about the survey's methodology and scope, and the online platform was set to mandate responses for every item while limiting each IP address to a single submission. Subsequently, data cleaning involved removing questionnaires with a completion duration of less than 5 min or those showing patterned responses. Additionally, attention check items (lie detection questions) were incorporated to verify the validity and sincerity of the responses.

## 3 Data analysis

SPSS (version 26.0) was employed to perform descriptive statistical analyses. Common method bias was assessed using Harman's single-factor test (Podsakoff et al., 2003).

LPA was performed to identify the most suitable latent profile solution for cybervictimization, utilizing Mplus (version 8.3). The optimal model solution was evaluated using standard indices, including the Akaike information criterion (AIC), Bayesian information criterion (BIC), Adjusted BIC (aBIC), Lo-Mendell-Rubin adjusted likelihood ratio test (LMRT), bootstrapped likelihood ratio test (BLRT), and entropy. Lower AIC, BIC, and aBIC values reflect superior model fit, while entropy values approaching 1 denote greater classification accuracy. Statistical significance in the LMRT and BLRT implies that the current k-class solution is significantly superior to the model with one fewer class (Burnham and Anderson, 2004). To explore differences across the emerging profiles, we applied analysis of variance (ANOVA) followed by Bonferroni tests, with partial eta squared (η2) calculated to signify effect sizes. In addition, differences in sex distribution across the profiles were examined using a chi-square test.

Network analysis was performed using *R* (version 4.5.1). A Gaussian Graphical Model (GGM) was estimated to capture the partial correlations between variables. To obtain a sparse estimation of the partial correlation matrix and prevent overfitting, we employed the EBICgLASSO function (the Extended Bayesian Information Criterion and the Graphical Least Absolute Shrinkage and Selection Operator) from the bootnet package (Epskamp et al., 2018). The tuning parameter γ was maintained at 0.5, aligning with standard methodological guidelines (Epskamp et al., 2018). For network visualization, the qgraph package was employed (Epskamp et al., 2012), wherein edges signify partial correlations; solid lines represent positive associations, dashed lines represent negative ones, and line thickness indicates the magnitude of the connection. Centrality indices were computed and transformed into Z-scores using the qgraph and networktools packages (McNally, 2016), focusing primarily on strength and expected influence. The strength represents the sum of the absolute weights of all edges connected to a node, reflecting its relative importance (Epskamp et al., 2012). The expected influence (EI) accounts for both positive and negative edge weights, capturing the cumulative impact of a node on the entire network (McNally, 2016). To evaluate network stability, we utilized a non-parametric bootstrap (1,000-time) through the bootnet package to calculate the 95% confidence intervals (CIs) for edge weights and the correlation stability (CS) coefficient, where values above 0.25 and 0.50 indicate moderate and high stability, respectively (Epskamp et al., 2018). The NetworkComparisonTest (NCT) package was applied to compare network models across different risk levels (low, moderate, and high cybervictimization groups) (van Borkulo et al., 2023). The network comparison analyses were based on permutation tests of global strength invariance and network structure invariance, with the significance level set at α = 0.05.

## 4 Result

### 4.1 Demographics

Among the 1,009 participants, 485 students were male (48.1%) and 524 were female (51.9%). In terms of educational level, 889 were undergraduate students (88.1%) and 120 were master's students (11.9%). The mean age of the participants was 21.26 (*SD* = 1.85) years. The total score on the Cyberbullying Victims' Behaviors Scale for College Students was 23.76 (*SD* = 7.16). For the subscales, the scores were 10.13 (*SD* = 3.73) for cyber verbal bullying, 4.89 (*SD* = 1.67) for hidden identity, and 8.73 (*SD* = 2.92) for cyber fabricate. The prevalence rate of cybervictimization among college students was 42.3%.

### 4.2 Test of common method bias

Harman's single-factor test revealed 17 factors with eigenvalues greater than 1. Furthermore, the first principal factor accounted for 18.3% of the total variance, which is below the threshold of 40%. This finding suggested that there was no significant common method bias in the present study.

### 4.3 Latent profile analysis for cybervictimization

LPA was conducted to identify distinct subgroup patterns of cybervictimization. The 12 individual items of the Cyberbullying Victims' Behaviors Scale were used as indicators to preserve item-level response patterns and minimize information loss. Latent profile models from one to four classes were sequentially estimated (Table 1). Although AIC, BIC, and aBIC decreased with increasing numbers of classes, the improvement in model fit became relatively small from the 3- to 4-class solution. The elbow plot (Supplementary Figure S1) further supported this pattern. Although LMRT and BLRT favored the 4-class solution, the additional class showed a response pattern highly similar to an existing moderate-severity class, suggesting limited substantive distinction between the two classes (Supplementary Figure S2). Moreover, the 3-class solution showed higher entropy and avoided the small subgroup sizes observed in the 4-class solution. Considering model fit, classification quality, and substantive interpretability, the 3-class solution was therefore selected as the optimal model. Based on the scoring patterns across the three classes, class 1 was labeled as the low cybervictimization group due to its consistently low scores. Class 2, defined as the moderate cybervictimization group, reflected intermediate levels of victimization, while class 3, representing the high cybervictimization group, exhibited the highest frequency of victimization. The proportions for the three groups were 48.3% (*n* = 486), 38.2% (*n* = 386), and 13.5% (*n* = 137), respectively (see Figure 2).

**Table 1:** Fitting indexes of latent profile model of cybervictimization in college students.

Model	AIC	BIC	aBIC	*p*LMRT	*p*BLRT	Entropy	Category probability(%)			
1	31,726.388	31,844.389	31,768.163	-	-	-	100	-	-	-
2	28,630.274	28,812.193	28,694.678	< 0.01	< 0.01	0.855	72.3	27.7	-	-
3	27,706.869	27,952.704	27,793.901	< 0.01	< 0.01	0.863	48.3	38.2	13.5	-
4	27,467.365	27,777.118	27,577.025	0.0296	0.0306	0.858	43.4	9.3	37.6	9.7

A1–A5 represent cyber verbal bullying victimization; A6–A7 correspond to hidden identity; and A8–A12 denote cyber fabricate.

**Figure 2:**
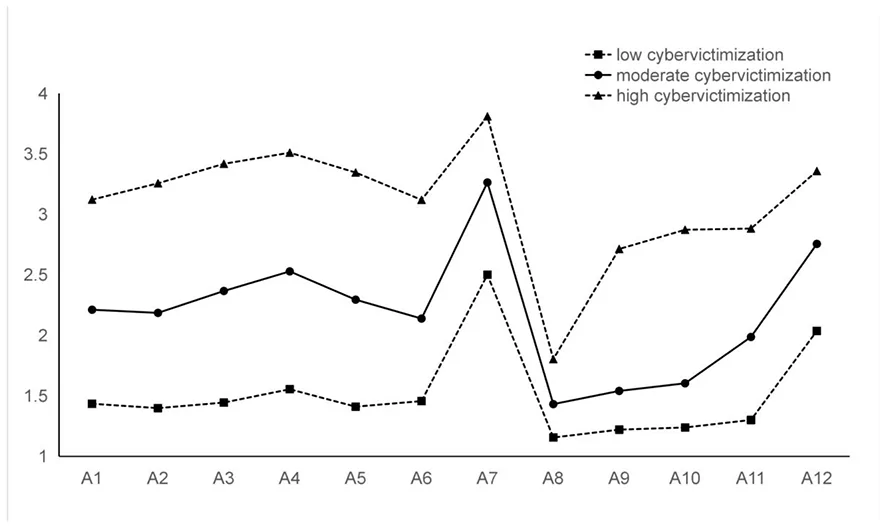
Distribution characteristics of three latent profiles of cybervictimization in college students.

### 4.4 Comparison of variables in each latent profile

Table 2 summarizes the results of the group comparisons examining possible differences in sociodemographic and psychological variables across the three latent profiles. One-way ANOVA indicated that depression, emotional regulatory self-efficacy, family functioning, school connectedness, peer victimization, maladaptive cognition in internet use, and online risky behaviors exhibited statistically significant differences among the groups (*F* = 13.124–198.272, *p* < 0 0.01), whereas no significant difference was found with respect to age. In addition, a chi-square test indicated that sex distribution differed significantly across the three profiles (χ^2^ = 9.21, *p* < 0.01). Together, these results indicate that the three latent profiles were well differentiated with respect to sex and the majority of the psychological variables examined.

**Table 2:** Univariate analysis of latent classes of cybervictimization in college students n (%).

Variables	Type 1 (*n* = 486)	Type 2 (*n* = 386)	Type 3 (*n* = 137)	*F/*χ2	Partial η2	Bonferroni *Post-hoc* test
Age	21.13 ± 1.88	21.31 ± 1.68	21.41 ± 1.87	2.599	0.005	-
Sex				9.210^*^	0.096	-
Male	211 (43.4)	197 (56.2)	77 (51.0)			
Female	275 (56.6)	189 (43.8)	60 (49.0)			
Depression	5.78 ± 4.11^a^	8.88 ± 4.29^b^	13.68 ± 4.45^c^	198.727^*^	0.283	a < b < c
Emotional regulatory self-efficacy						
Expressing positive affect	16.31 ± 2.85^a^	15.80 ± 2.86^b^	14.39 ± 3.45^c^	23.461^*^	0.044	a > b > c
Managing distress	13.14 ± 3.47^a^	11.55 ± 3.19^b^	11.31 ± 3.52^c^	30.150^*^	0.056	a > b, a > c
Managing anger	13.02 ± 3.69^a^	11.49 ± 3.23^b^	11.16 ± 3.63^c^	27.046^*^	0.051	a > b, a > c
Family functioning	23.05 ± 5.17^a^	20.75± 5.63^b^	18.98 ± 6.26^c^	36.987^*^	0.068	a > b > c
School connectedness						
School belonging	11.21 ± 2.48^a^	10.43 ± 2.81^b^	9.73 ± 3.06^c^	19.417^*^	0.037	a > b > c
Teacher support	11.51 ± 2.30^a^	10.53 ± 2.63^b^	9.80 ± 2.75^c^	31.901^*^	0.059	a > b > c
Peer support	15.68 ± 3.19^a^	15.01 ± 3.46^b^	14.06 ± 3.91^c^	13.124^*^	0.024	a > b > c
Peer victimization						
Verbal victimization	5.63 ± 2.18^a^	6.99 ± 2.43^b^	8.93 ± 2.59^c^	115.255^*^	0.186	a < b < c
Relational victimization	5.07 ± 2.22^a^	6.53 ± 2.59^b^	8.64 ± 2.73^c^	123.677^*^	0.197	a < b < c
Physical victimization	3.94 ± 1.68^a^	4.96 ± 2.16^b^	6.39 ± 2.88^c^	81.324^*^	0.139	a < b < c
Maladaptive cognition in internet use						
Online comfort	16.12 ± 3.79^a^	17.08 ± 3.51^b^	17.72 ± 3.58^c^	13.537^*^	0.026	a < b, a < c
Diminished impulse control	14.88 ± 3.94^a^	16.80 ± 3.48^b^	18.16 ± 3.78^c^	52.855^*^	0.095	a < b < c
Escape and withdrawal	12.67 ± 3.58^a^	14.07 ± 2.93^b^	15.34 ± 2.70^c^	43.849^*^	0.080	a < b < c
Online risky behaviors						
Social risk	21.52 ± 5.37^a^	23.88 ± 4.30^b^	24.51 ± 4.88^c^	33.956^*^	0.063	a < b, a < c
Entertainment risk	12.74 ± 4.44^a^	15.87 ± 4.61^b^	17.47 ± 4.74^c^	82.999^*^	0.142	a < b < c
Contact risk	5.35 ± 2.19^a^	6.47 ± 2.39^b^	7.48 ± 2.47^c^	54.432^*^	0.098	a < b < c
Transaction risk	6.79 ± 2.65^a^	8.17 ± 2.95^b^	9.88 ± 2.84^c^	72.880^*^	0.126	a < b < c
Management risk	8.26 ± 2.73^a^	10.12 ± 2.87^b^	11.25 ± 3.17^c^	79.927^*^	0.137	a < b < c

Type 1 = low cybervictimization, Type 2 = moderate cybervictimization, Type 3 = high cybervictimization. a, b, c showed the results of the Bonferroni *post-hoc* tests and there is a statistically significant between two. e.g., for depression, “a < b < c” means that class 1 has a mean score that is lower than class 2 and 3. ^*^P < 0.01.

### 4.5 Network analysis of factors related to cybervictimization

The network structure of factors associated with cybervictimization among college students was illustrated in Figure 3. The network consisted of 19 nodes with 95 non-zero edges. The average edge weight across the entire network was 0.09, and the mean predictability of the 19 nodes was 0.471, with school belonging (SB) showing the highest predictability (*R*^2^ = 0.689). Within the network, the strongest association was observed between teacher support (TS) and SB (Weight = 0.612). Furthermore, both TS and family functioning (FF) showed negative edges with depression (DEP), (Weight = 0.119/0.117).

**Figure 3:**
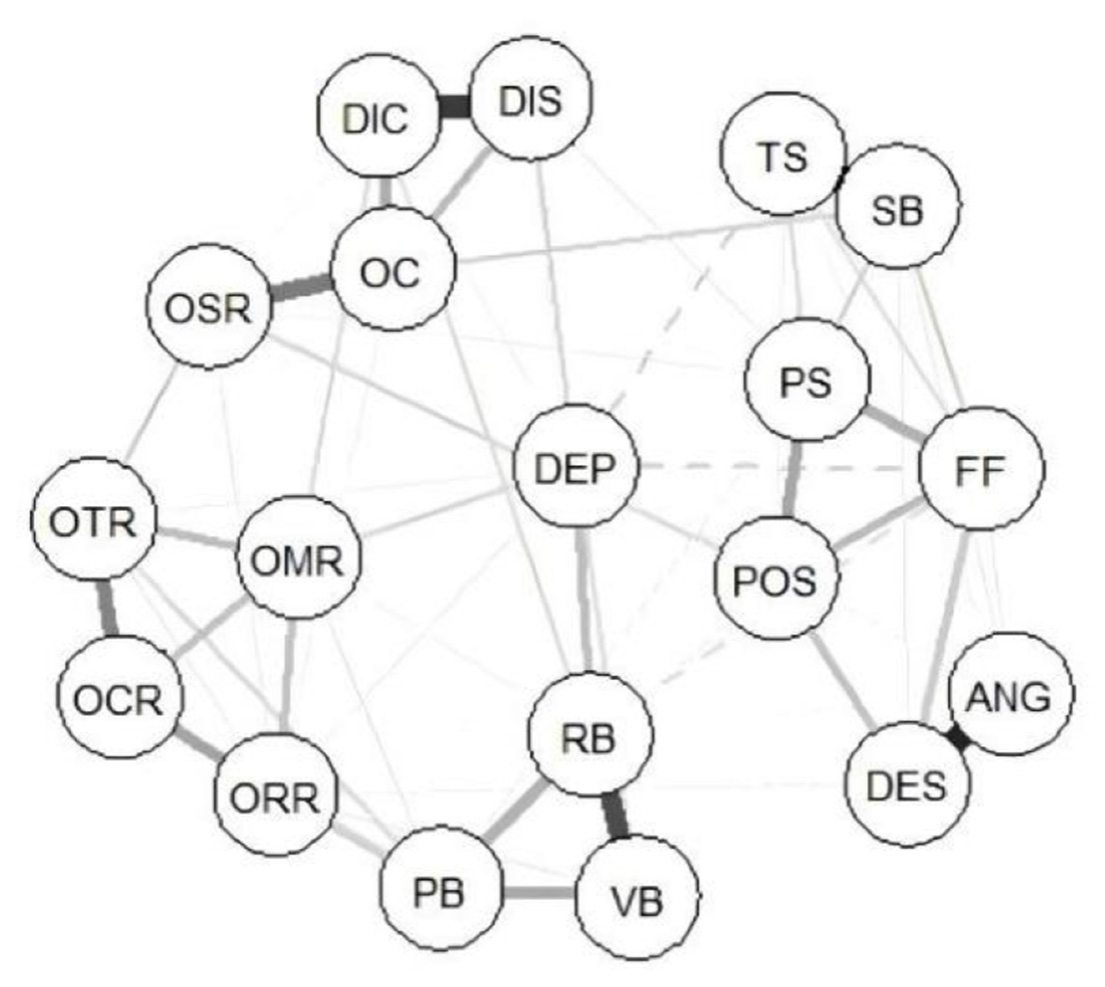
Network of overall correlated factors for cybervictimization among college students. DEP, Depression; FF, Family Functioning; OSR, Online Social Risk; ORR, Online Entertainment Risk; OCR, Online Contact Risk; OTR, Online Transaction Risk; OMR, Online Management Risk; VB, Verbal Victimization; RB, Relational Victimization; PB, Physical Victimization; ANG, perceived self-efficacy in managing anger; DES, perceived self-efficacy in managing distress; POS, perceived self-efficacy in expressing positive affect; SB, School belonging; TS, Teacher Support; PS, Peer Support.

Figure 4 illustrates the standardized centrality indices of the network. SB exhibited the highest strength centrality and expected influence (Strength = 2.125, EI = 1.920), followed by relational victimization (RB) (Strength = 1.624, EI = 0.577).

**Figure 4:**
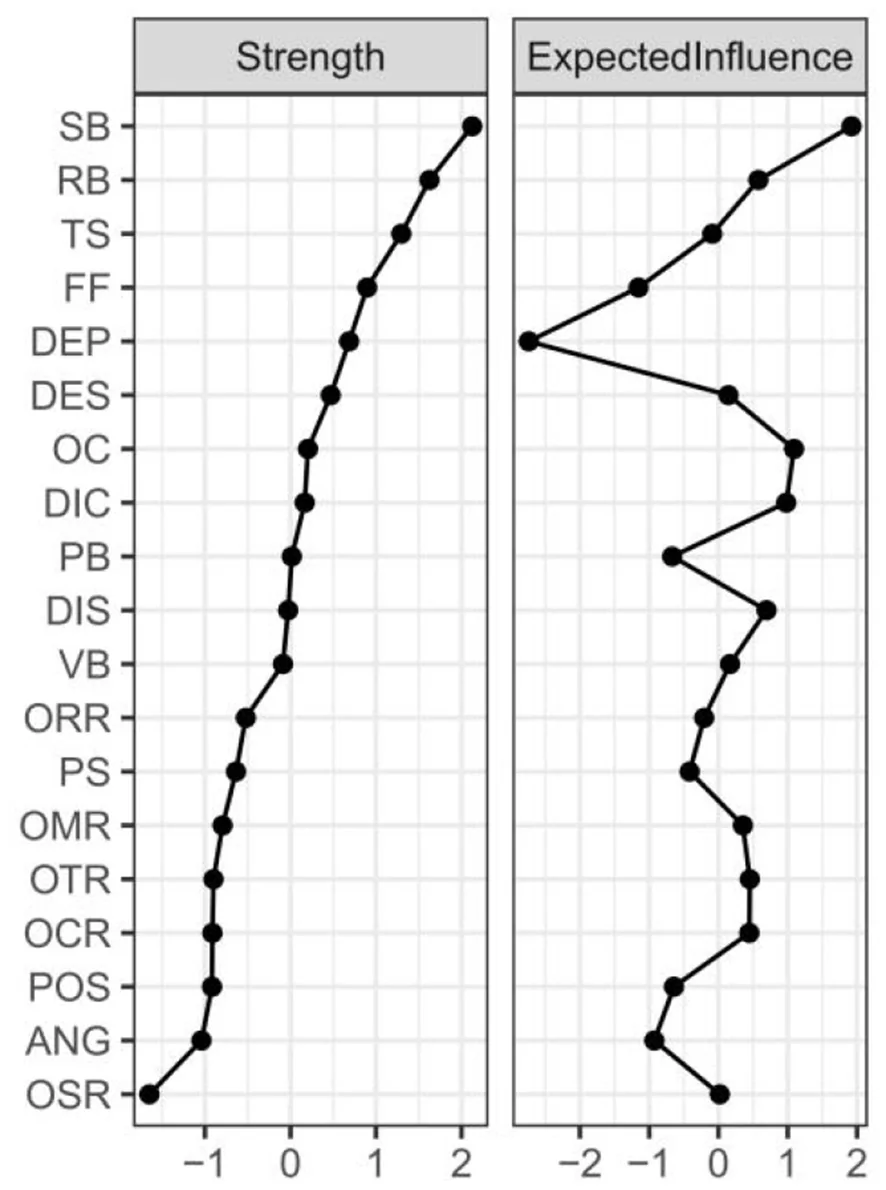
Standardized evaluation of network centrality.

A non-parametric bootstrap procedure (1,000 times) was employed to estimate the 95% *CI* for edge weights and to compare differences in centrality indices (see Figure 5). The relatively narrow 95% *CI* for the edge weights indicated that the estimations were sufficiently accurate. Furthermore, the CS coefficients for Strength and EI were 0.67 and 0.60, respectively. Both values exceeded the recommended threshold of 0.50, demonstrating that the network architecture possessed high stability (see Figure 6).

**Figure 5:**
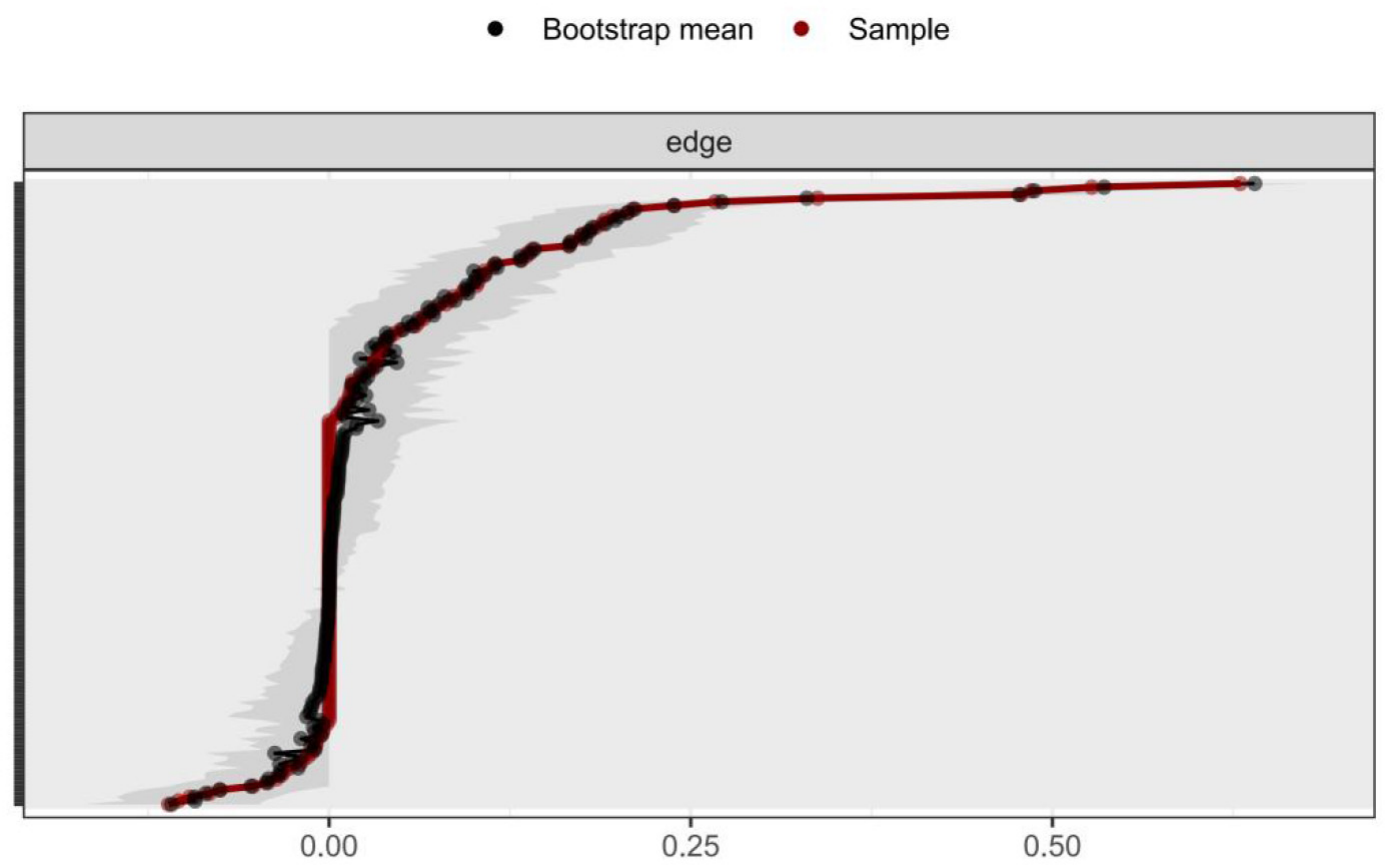
Accuracy verification of edge.

**Figure 6:**
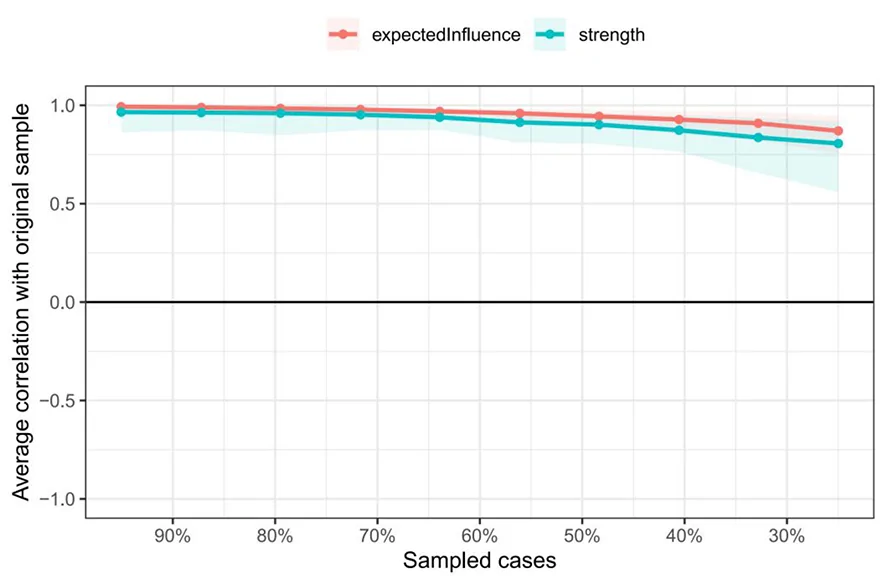
Stability assessment of centrality coefficients.

### 4.6 Differences in network structures across latent profiles

Figure 7 illustrates the network structure of related psychosocial factors among different subgroups. The low, moderate, and high cybervictimization groups consisted of 79, 79, and 67 non-zero edges, respectively, with corresponding mean predictability values of 0.441, 0.378, and 0.517 respectively. The average absolute edge weights for the three groups were 0.096, 0.090, and 0.116, indicating variations in connectivity strength across the profiles. In all groups, the strongest association was consistently observed between TS and SB (Weight: 0.505~0.585). Further analysis revealed group specific patterns: in the moderate cybervictimization group, a negative edge was found between physical bullying (PB) and efficacy in expressing positive emotions (POS) (Weight: −0.126). In the high cybervictimization group, a significant negative edge emerged between relational bullying (RB) and FF (Weight: −0.129). These findings highlight distinct structural differences among the network models of the three groups.

**Figure 7:**
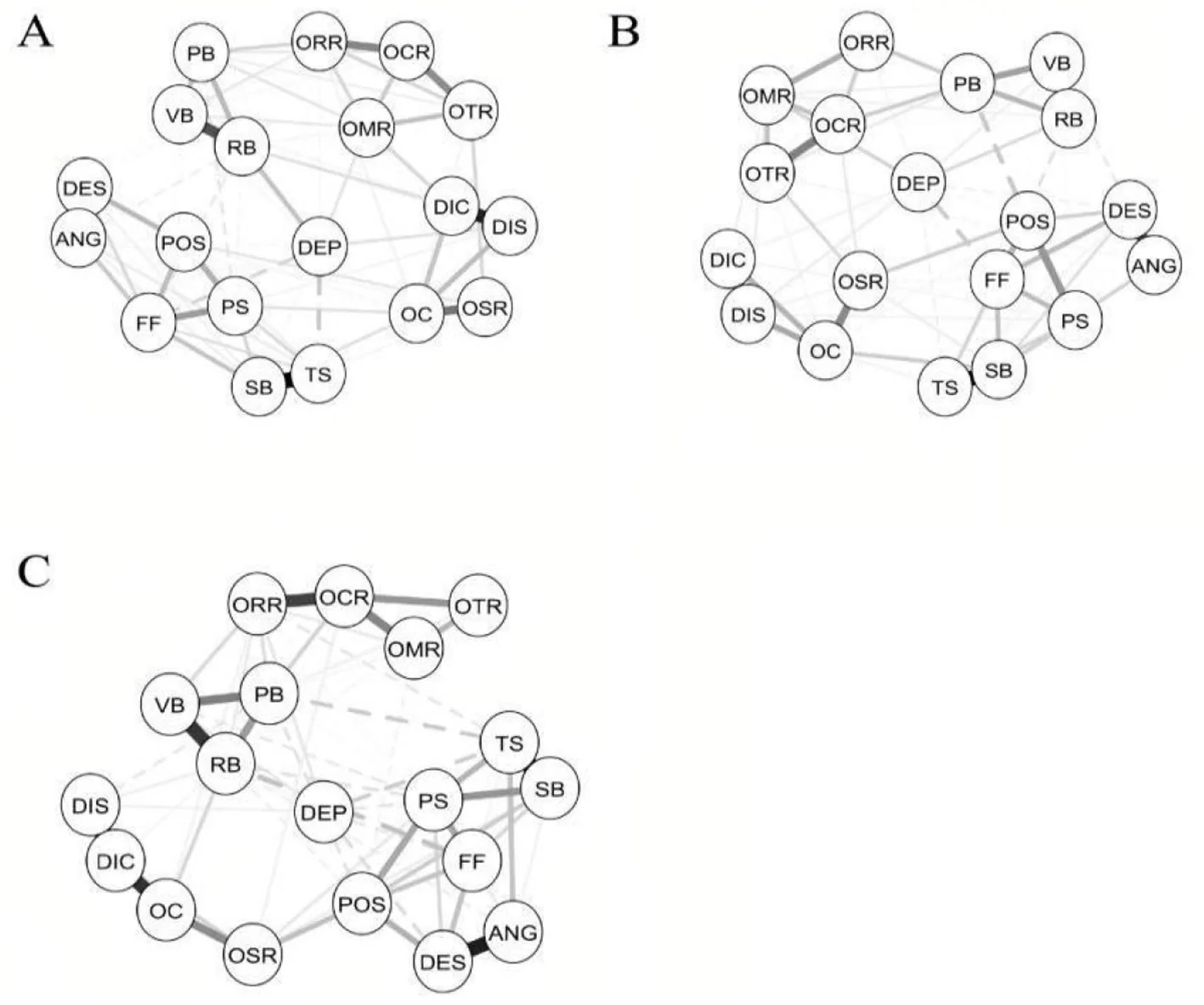
Network of factors associated with cybervictimization subgroups among college students. **(A)** low cybervictimization, **(B)** moderate cybervictimization, **(C)** high cybervictimization.

The features of node centrality for each subgroup are presented in Figure 8. Stability tests showed that the CS-coefficients for strength and expected influence were above 0.25 across most groups, with the exception of expected influence in the high-victimization group (CS = 0.204), which fell below the recommended threshold, indicating limited stability. Accordingly, EI was not interpreted for the high-victimization subgroup, and subsequent interpretations of node centrality were based on strength centrality, which demonstrated adequate stability. With this in mind, the results indicated that SB remained the core node across all three networks (type 1: Strength/EI = 1.593/1.333; type 2: Strength/EI = 2.616/2.51; type 3: Strength/EI = 1.109/1.197). Online comfort (OC) exhibited relatively high strength and expected influence in both the low and moderate cybervictimization groups (type1: Strength/EI = 0.481/1.041, type2: Strength/EI = 0.389/0.931). In the high cybervictimization group, deficient impulse control (DIC) and online contact risk (OCR) showed relatively high strength (1.060/0.730). The NCT revealed no significant differences in global network strength between any pairs (low vs. moderate: *S* = 0.493, *p* > 0.05, low vs. high: *S* = 0.145, *p* > 0.05, and moderate vs. high: *S* = 0.623, *p* > 0.05). Regarding global network structure, no significant differences were found between the low and moderate groups (*M* = 0.177, *p* > 0.05) or between the moderate and high groups (*M* = 0.266, *p* > 0.05); however, the low and high groups differed significantly (*M* = 0.268, *p* < 0.05). Given that the network structure invariance test aggregates evidence across all edges, we conducted *post-hoc* edge invariance tests with Holm correction for multiple comparisons across all 171 possible edges to examine whether this structural difference could be attributed to specific network connections. After correction, no individual edge difference reached statistical significance (all corrected *p* > 0.05). The smallest corrected *P*-values were observed for the edges connecting online comfort with deficient impulse control (*p* = 0.342, *E* = 0.268) and with relational bullying (*p* = 0.849, *E* = 0.838); however, these differences did not survive multiple comparison correction.

**Figure 8:**
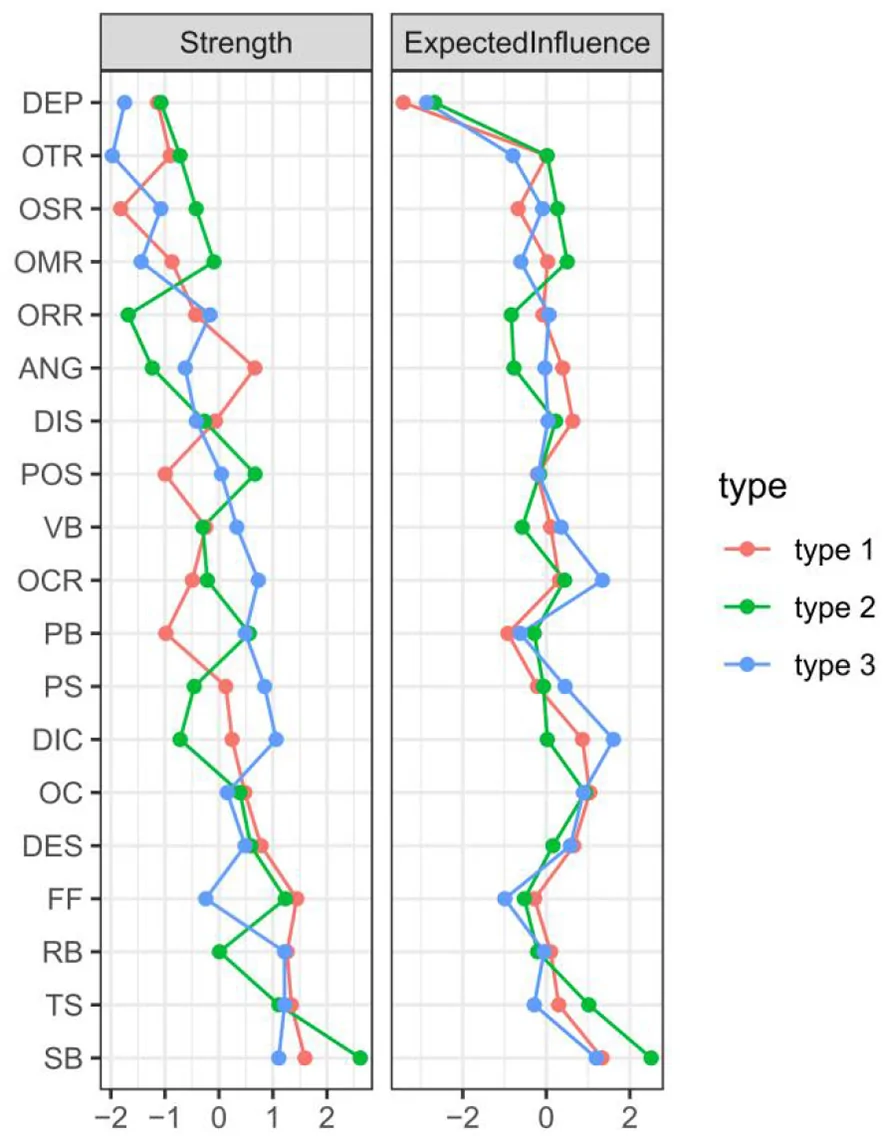
Standardized evaluation of network centrality in three groups. type 1 = low cybervictimization, type 2 = moderate cybervictimization, type 3 = high cybervictimization.

### 4.7 Sensitivity analysis: network structure comparison between male and female participants

To further examine whether sex confounded the subgroup network comparisons reported above, we conducted a Supplementary analysis comparing network structures between male and female participants using the same estimation method (EBICglasso, γ = 0.5). Results showed no significant difference in global strength (*S* = 0.112, *p* = 0.734). Network structure invariance reached marginal significance (*M* = 0.184, *p* = 0.040). *Post-hoc* edge invariance tests were then conducted with Holm correction for multiple comparisons across all 171 possible edges. No individual edge difference reached statistical significance after correction (all corrected *p* > 0.05; minimum corrected *p* = 0.342).

At the node level, SB, RB, and OC emerged as the top three central nodes in both the male and female networks, (see Figure 9). Some sex specific patterns were nonetheless observed: among male participants, SB showed the highest values on both centrality indices, whereas among female participants RV showed the highest node strength, with SB remaining the leading node in expected influenc, (see Table 3). Both networks demonstrated adequate stability, with CS-coefficients for strength and expected influence exceeding the recommended threshold of 0.50.

**Figure 9:**
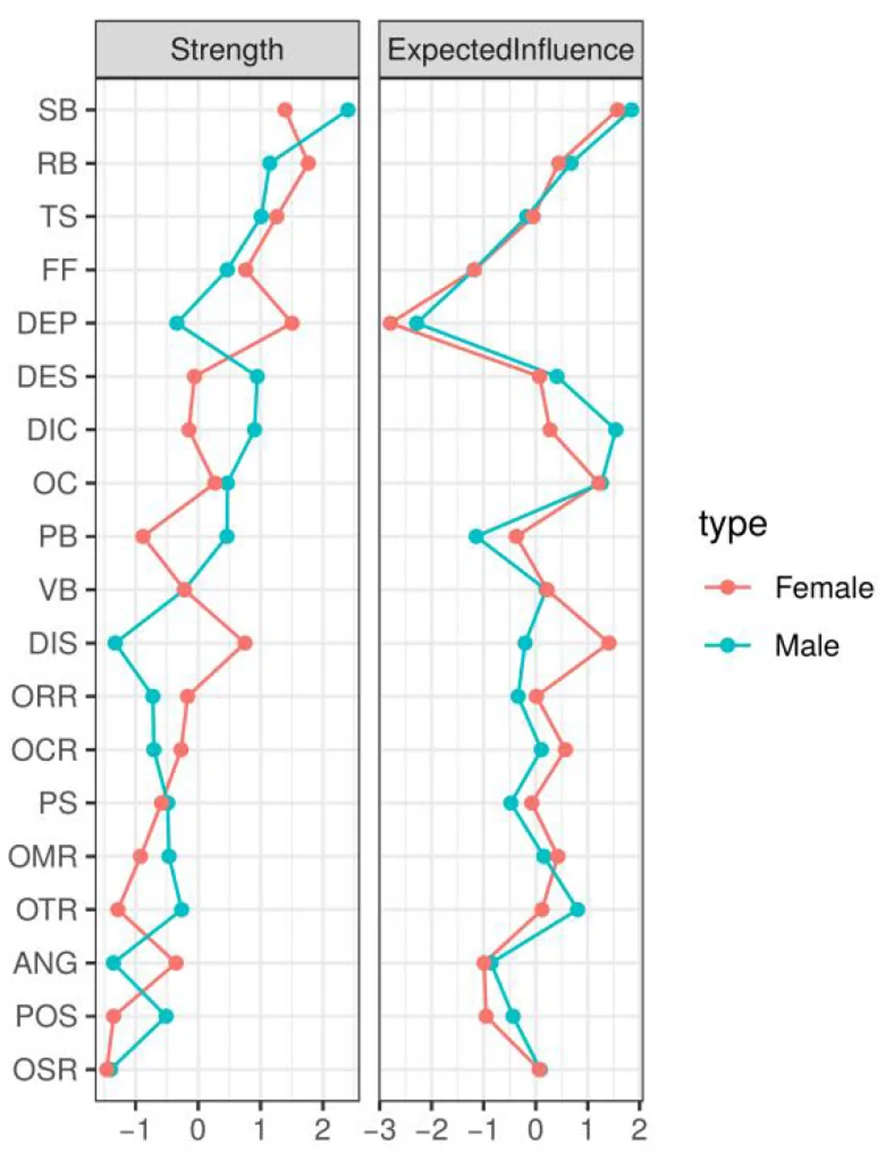
Standardized evaluation of network centrality in male and female networks.

**Table 3:** Centrality indices of primary nodes in male and female networks.

Node	Male		Female	
	Strength	EI	Strength	EI
SB	1.153	1.051	1.105	1.029
RB	1.027	0.865	1.154	0.806
OC	0.959	0.959	0.958	0.958

## 5 Discussion

The present study identified three subgroups of cybervictimization, including the low cybervictimization group (48.3%), the moderate cybervictimization group (38.2%), and the high cybervictimization group (13.5%). The results revealed significant differences across subgroups in item-level scores on the cybervictimization questionnaire, with scores on the hidden identity dimension being significantly higher than those on the other dimensions. One possible explanation may relate to the anonymity afforded by online environments. Anonymity can weaken the connection between aggressive behavior and one's real-world identity, thereby reducing concerns about social evaluation and identity exposure and potentially weakening moral self-restraint. Such conditions may make individuals more likely to engage in aggressive behaviors when the perceived risk of real-world consequences is reduced (Barlett et al., 2021). The distribution of the identified profiles also exhibited a pyramid-like pattern, with subgroup sizes decreasing as the severity of cybervictimization increased. This pattern is broadly consistent with previous studies using LPA to examine subgroups of cyberbullying victimization among college students (Aparisi et al., 2023), suggesting that severe cybervictimization experiences may be relatively uncommon in this population. Notably, the characterization of the profiles identified in the present study differed to some extent from that reported by Zhan et al. (2026), whose subgroup labels more directly reflected specific forms of cybervictimization. Such discrepancies may partly reflect differences in regional contexts between the two samples, as variations in online environments and sociocultural contexts across regions may shape the manifestation and co-occurrence patterns of different forms of cybervictimization. Overall, these findings suggest that cybervictimization experiences among college students are heterogeneous, with individuals differing not only in the severity but also potentially in the forms of victimization they experience. This heterogeneity provides a rationale for further examining whether the patterns of associations among psychosocial factors also differ across cybervictimization profiles.

Network analysis further revealed cross-level associations among psychosocial factors related to cybervictimization. For example, teacher support was associated with school belonging and depressive symptoms, whereas family functioning was associated with relational victimization and depressive symptoms. These associations are consistent with the ecological systems perspective (Bronfenbrenner, 1979), which emphasizes interactions between individuals and their social environments, suggesting that cybervictimization among college students should be understood within the interconnected contexts of individual, family, and school factors rather than in terms of isolated factors (Dai et al., 2025).

School belonging emerged as a central node in both the overall network and all three subgroup networks, suggesting that it may represent an important protective factor within the psychosocial system associated with cybervictimization. Its relatively high predictability further suggests that school belonging is closely embedded within students' broader psychosocial environment. Notably, although family functioning remained a substantively connected node within the network, its centrality was less pronounced than that of school belonging. According to emerging adulthood theory, individuals at this developmental stage gradually transition from dependence on their family of origin toward greater autonomy and expanded social relationships (Twenge, 2008). During this period, daily sources of interpersonal support, identity development, and emotional connection may become increasingly embedded in the university context, including dormitory relationships, peer interactions, extracurricular activities, and student-teacher interactions (Scharfe et al., 2017). Thus, for university students, the campus environment may partially assume some of the functions of belonging and support traditionally provided by the family. This finding suggests the potential importance of strengthening social connection and support within the university environment for students at this developmental stage.

Beyond school belonging as a common central node, the subgroup networks also showed some differences according to the level of cybervictimization. In the low and moderate cybervictimization groups, online comfort consistently showed relatively high centrality. Online comfort represents one aspect of maladaptive Internet-use cognition and primarily reflects the extent to which individuals feel at ease and experience reduced anxiety during online social interactions compared with offline contexts (Davis et al., 2002). Previous research has suggested that individuals with greater online comfort tend to be more familiar with the online environment (Caplan, 2003), while more frequent and intensive online activity may increase opportunities for exposure to negative online interactions (Gainsbury et al., 2019). This pattern is consistent with the routine activity perspective, which suggests that patterns of online activity and the extent of target exposure may influence the risk of victimization (Lee et al., 2026). Thus, the prominence of online comfort in the low and moderate cybervictimization groups may reflect the link between familiarity with the online environment, greater exposure through online activities, and cybervictimization. These findings suggest that future interventions for these groups could consider promoting more adaptive cognitions regarding Internet use and improving students' awareness of potential risks associated with online exposure.

In contrast, deficient impulse control and online contact risk showed relatively high node strength in the high cybervictimization group. These two factors reflect, respectively, difficulties in regulating patterns of Internet use and controlling online behavior (Davis et al., 2002), and the extent to which individuals interact online with strangers or potentially risky individuals (Savoia et al., 2021). This difference from the prominence of online comfort in the low and moderate cybervictimization groups may reflect differences in the online experiences and behavioral exposure patterns associated with different levels of cybervictimization. Low and moderate levels of cybervictimization may more often involve relatively occasional negative online experiences, whereas high levels of cybervictimization may involve more frequent or repeated victimization experiences. Under such circumstances, persistent engagement in high-risk online behaviors may be associated with more frequent exposure to potentially harmful situations, which correspondingly relates to greater opportunities for further victimization. The repeat victimization perspective suggests that experiencing victimization may be associated with an elevated risk of subsequent victimization, while persistent risk-related behaviors and lifestyles may contribute to the maintenance of such exposure (Turanovic and Pratt, 2014). Thus, the relatively high centrality of online contact risk and deficient impulse control in the high cybervictimization group may indicate the greater relevance of behavioral exposure and difficulties in behavioral regulation at higher levels of cybervictimization. Previous research has similarly reported associations between impulsivity, high-risk online behaviors, and cybervictimization (Álvarez-García et al., 2019). Thus, the relatively high centrality of online contact risk and deficient impulse control in the high group suggests that behavioral exposure and regulation become more relevant at greater severity, with intervention targets correspondingly shifting from cognitive appraisal. Nonetheless, given the relatively small sample size of the high cybervictimization group, this interpretation should be treated as exploratory.

The NCT showed no significant differences in global network strength across the groups, suggesting that increasing levels of cybervictimization severity were not accompanied by stronger overall connectivity among the variables. Notably, no significant difference in overall network structure was observed between the moderate cybervictimization group and either the low or high group, and a significant structural difference emerged only between the low and high groups. Although this overall structural difference reached significance, *post-hoc* pairwise edge comparisons did not identify any individual edge that remained significant after correction for multiple comparisons, suggesting that it likely reflects the cumulative effect of many small, distributed edge-level discrepancies rather than one or a few dominant connections. This pattern suggests that increasing levels of cybervictimization may not be accompanied by a linear change in the associative structure of psychosocial variables, but rather by a degree of non-uniformity. A similar non-uniform pattern of between-group structural differences has been reported in multi-group comparisons of psychopathology symptom networks, where not all adjacent severity groups showed significant structural differences (Imperiale et al., 2021). Direct empirical evidence explaining why structural differences emerged only between the two extreme groups is currently lacking. One possible explanation, drawing on the allostatic load framework (McEwen and Stellar, 1993), is that lower levels of cybervictimization may not exceed the adaptive capacity of individuals' psychological regulatory resources, whereas greater cumulative victimization may increase psychological regulatory demands and, in turn, be associated with more pronounced changes in network structure (Guidi et al., 2020). This interpretation remains speculative in the specific context of cybervictimization and requires testing in future studies designed to directly assess regulatory capacity across victimization levels. Alternatively, this result may also be related to how cybervictimization levels were grouped: the gap in victimization intensity between the low and high groups is relatively large, which may make structural differences easier to detect. Future research should therefore use larger samples to develop more fine-grained subgroupings of cybervictimization severity, in order to better pinpoint the severity threshold at which this non-uniform reorganization occurs and to rule out grouping effects as an alternative explanation.

Given that previous research has documented sex differences in both the prevalence and correlates of cybervictimization (Zsila et al., 2019), we conducted exploratory gender-stratified network comparisons. Although the overall connectivity strength of the male and female networks was comparable, and no clear sex differences in individual edges were identified after correction for multiple comparisons, the ranking of central nodes showed some differences between the two networks. Notably, relational victimization exhibited the highest node strength in the female network. This finding may be related to sex differences in patterns of exposure to relational victimization (Casper et al., 2020). Longitudinal research has shown that, compared with boys, girls are more likely to follow trajectories characterized by persistent moderate levels of victimization (Orpinas et al., 2015). More sustained experiences of relational victimization may be associated with broader connections with depression, social support, and other psychosocial factors related to cybervictimization (An et al., 2024; Crick and Nelson, 2002; Wang et al., 2024), potentially contributing to its higher connectivity within the female network. Therefore, relational victimization may play a relatively prominent role in the psychosocial network associated with cybervictimization among female students and warrants further attention in future research and practice.

Overall, by integrating latent profile analysis and network analysis, the present study further advances the understanding of heterogeneity in cybervictimization among college students. The latent profile analysis first showed that college students do not experience cybervictimization in a homogeneous manner but can be characterized by distinct levels of victimization. Building on this finding, the network analyses further revealed that psychosocial factors were not organized in entirely the same way across groups with different levels of cybervictimization. Specifically, school belonging showed high centrality across all profiles, suggesting that some psychosocial factors may retain relatively consistent importance across different levels of victimization. At the same time, differences in central nodes across profiles, together with the structural differences observed between the low- and high cybervictimization groups, suggest that heterogeneity in cybervictimization may extend beyond differences in victimization severity to encompass variations in the organization of associated psychosocial factors. Therefore, examining cybervictimization-related factors solely at the whole-sample level may not fully capture potential differences across subgroups. Integrating person-centered latent profile analysis with variable-centered network analysis provides a complementary perspective for understanding the complexity of cybervictimization from both individual heterogeneity and patterns of associations among psychosocial factors.

Importantly, these findings also provide preliminary directions for future prevention and support research. Given the cross-sectional design, the following implications should be considered provisional and require further confirmation through longitudinal and intervention research. School belonging showed relatively high centrality across all three cybervictimization groups. This shared characteristic suggests that, despite differences in the severity of cybervictimization, psychosocial resources related to the university environment may represent a common area warranting attention across groups. Future research could therefore examine the potential value of university-based support approaches aimed at strengthening students' social connections and sense of belonging. For example, peer support programs, opportunities for student participation, and activities promoting positive student–teacher and peer relationships may represent potential directions for further evaluation. Beyond this shared characteristic, the low and moderate cybervictimization groups also exhibited a relatively consistent pattern, with online comfort emerging as a prominent node in both networks. This finding suggests that, among students experiencing relatively lower levels of cybervictimization, the relationship between subjective comfort in the online environment and actual exposure to online risks may warrant further attention. Potential directions for support may include helping students recognize that feeling comfortable in online interactions does not necessarily imply protection from potential online risks and enhancing their ability to identify potentially high-risk online situations. In contrast, the high cybervictimization group displayed a different pattern, with online contact risk and deficient impulse control emerging as more prominent nodes. This pattern suggests that future support research involving students experiencing more severe cybervictimization may need to pay greater attention to behavioral exposure and behavioral self-regulation. Future intervention studies could explore support approaches that integrate online safety education, the identification of high-risk online contacts, and behavioral self-regulation training. For example, scenario-based exercises could be used to help students identify potentially risky online interactions and strengthen their capacity for behavioral regulation in complex online social situations, thereby exploring possible ways to promote safer patterns of Internet use.

## 6 Limitations and future directions

This study has several limitations. First, the utilizing of a cross-sectional design restricts our capacity to establish causal links between the examined variables. Future studies may address this limitation by employing longitudinal designs. Second, the sample was recruited exclusively from three comprehensive universities in Shandong Province, which may limit the representativeness and generalizability of the findings. As an economically developed coastal province, Shandong may differ from other regions in terms of internet infrastructure, digital literacy, and campus culture, which may shape patterns of cybervictimization. Moreover, vocational colleges and other types of higher-education institutions were not included. Therefore, the identified latent profiles and network structures may not fully represent the diversity of cybervictimization experiences among Chinese college students. Future research should recruit more geographically and institutionally diverse samples across different regions, university types, and urban/rural backgrounds. Third, although the Chinese version of the Cyberbullying Victimization Scale used in this study has demonstrated good psychometric properties among Chinese college students, its three-dimensional structure was originally derived from a Turkish cultural context. As such, it may not fully capture certain culturally specific manifestations of cybervictimization among Chinese college students, such as group-based exclusion on widely used domestic social platforms or indirect mockery conveyed through internet memes and popular online slang. Future research may adopt qualitative or mixed-method approaches to explore indigenous dimensions of cybervictimization within the Chinese cultural context and further refine the measurement instrument. Fourth, the temporal frameworks of the measures were not fully aligned. Cybervictimization was assessed over the past year, whereas depressive symptoms were assessed over the past two weeks. Although these time frames are, respectively, common practice for capturing relatively low-frequency victimization events and recent depressive symptomatology, their discrepancy may introduce recall bias for cybervictimization and complicate inferences about the observed associations: for instance, associations may be underestimated if the psychological impact of earlier victimization had already subsided by the time depressive symptoms were assessed, or overestimated if currently depressed individuals recalled past victimization more negatively. In addition, the cross-sectional design prevents the temporal ordering among cybervictimization, depressive symptoms, and other psychosocial factors from being established. Future longitudinal research employing more temporally aligned or explicitly sequenced assessment windows is needed to clarify these temporal relationships and disentangle their causal ordering. Fifth, the relatively small sample size of the high cybervictimization group (*n* = 137) may limit the stability and precision of its subgroup network estimates. This concern is reflected in the CS-coefficient for expected influence, which was 0.204 and below the commonly referenced threshold of 0.25, indicating limited stability of this centrality estimate. Accordingly, findings concerning the network characteristics of the high cybervictimization group, particularly expected influence, should be interpreted as preliminary and with caution. Future studies should recruit larger samples of highly victimized individuals to further evaluate the stability and generalizability of these findings. Finally, although sensitivity analyses were conducted to assess the potential influence of age and sex, neither variable was formally incorporated as a covariate in the subgroup network estimation. Age did not differ significantly across the three latent profiles, whereas the overall distribution of sex differed significantly across profiles, although none of the pairwise comparisons remained significant after correction for multiple comparisons. Network comparison by sex revealed no significant edge differences after Holm correction, although the test of global network structure invariance indicated a significant difference. These findings provide preliminary evidence regarding the potential influence of demographic characteristics, but they do not constitute formal covariate adjustment. Therefore, the possibility that age and sex may have influenced the subgroup network estimates cannot be completely excluded. Future studies should incorporate these demographic characteristics as covariates in network estimation to more rigorously account for their potential influence.

## 7 Conclusion

This study identified three latent profiles of cybervictimization among college students, characterized by low, moderate, and high levels of victimization. School belonging showed relatively high centrality across all profiles, whereas online comfort was more prominent in the low and moderate cybervictimization groups, and online contact risk and deficient impulse control were more prominent in the high cybervictimization group. Structural differences were observed between the low and high cybervictimization groups, although global network strength did not differ significantly across groups. By integrating latent profile and network analyses, this study provides a complementary perspective on heterogeneity in cybervictimization and its associated psychosocial patterns. These findings offer preliminary directions for future research on subgroup-informed prevention and support, which require validation in larger longitudinal and intervention studies.

## Data Availability

The raw data supporting the conclusions of this article will be made available by the authors, without undue reservation.
